# Configuration of the Volatile Aromatic Profile of Carob Powder Milled From Pods of Genetic Variants Harvested at Progressive Stages of Ripening From High and Low Altitudes

**DOI:** 10.3389/fnut.2021.789169

**Published:** 2021-12-15

**Authors:** Chrystalla Antoniou, Angelos C. Kyratzis, Georgios A. Soteriou, Youssef Rouphael, Marios C. Kyriacou

**Affiliations:** ^1^Department of Vegetable Crops, Agricultural Research Institute, Nicosia, Cyprus; ^2^Department of Agricultural Sciences, University of Naples Federico II, Portici, Italy

**Keywords:** isobutyric acid, maturity, propanoate esters, volatile organic compounds, volatilome, headspace gas chromatography mass spectrometry

## Abstract

Carob powder is increasingly valued as a substitute for cocoa and as a flavor-enhancing component of processed foods. However, little is known about the impact of preharvest factors such as fruit maturity, genotype and altitude on its volatile organic compounds (VOCs) composition. The current study examined the VOCs composition of powder milled from pods of two genotypes cultivated at 15 and 510 m altitude and harvested at six progressive stages of maturity, ranging from fully developed immature green (RS1) to late ripe (RS6). Fifty-six VOCs categorized into acids, esters, aldehydes, ketones, alcohols, furans, and alkanes were identified through HS-SPME GC-MS analysis. Maturity was the most influential factor, followed by altitude and least by genotype. Aldehydes and alcohols correlated positively (*r* = 0.789; *p* < 0.001), both accumulated in immature carobs and decreased with progressive ripening, resulting in the attenuation of green grassy aroma. Conversely, acids increased with ripening and dominated the carob volatilome at full maturity, correlating negatively with aldehydes and alcohols (*r* = −0.835 and *r* = −0.950, respectively; *p* < 0.001). The most abundant VOC throughout ripening (17.3-57.7%) was isobutyric acid, responsible for the characteristic cheesy-acidic-buttery aroma of carob powder. The pleasurable aroma detected at the immature stages (RS2 and RS3) was traced to isobutyrate and methyl isobutyrate esters, rendering unripe green carob powder a potential admixture component for improving the aroma of novel food products. Lower altitude favored the accumulation of acids linked to less pleasant aroma, whereas isobutyric acid was more abundant at higher altitude. This constitutes a significant indication that higher altitude enhances the characteristic carob-like aroma and sensory quality of carob powder.

## Introduction

The carob (*Ceratonia siliqua* L.) is an underutilized crop that belongs to the *Fabaceae* family, growing naturally and cultivated non-intensively for centuries in the Mediterranean basin. The revived interest in carob cultivation observed in recent years is driven by its low input requirements, its resilience to marginal soils and abiotic stress factors (1), and the particular sensory profile and multiple functional properties of the carob fruit (pod) that benefit human health (2). The carob pod is a multipurpose foodstuff consisting of a sugar-dense pulp (90% w/w) and the seeds (10% w/w), the latter being considered industrially most valuable as the source of the locust bean gum (LBG) food stabilizer (3). The kibbled carob pulp is a low-cost byproduct of the pod milling process that is utilized by the food industry to produce various high value functional products such as molasses (carob syrup), carob juice and powder (flour and fiber).

Carob powder is generally prepared from roasted and unroasted coarse carob kibbles milled to a granulometry <2.0 mm and bearing an intensely caramelized aroma (4). It is increasingly valued as a substitute for cocoa and finds its way as an ingredient in a wide range of processed foods (e.g., morning cereals) due to its low fat (0.6%) and high dietary fiber content (40%) (5, 6). Moreover, carob powder is a great source of sugars and bioactive secondary metabolites such as polyphenols (e.g., tannins, catechins, gallotanins, and gallic acid) and soluble peptides (7, 8) associated with numerous health-promoting properties ranging from radical scavenging to antiproliferative action on colon cancer and hepatocellular carcinoma, the control of diarrhea symptoms, the lowering of LDL cholesterol and antidiabetic effects (2, 9, 10).

Apart from its eminent nutritional and nutraceutical properties, carob powder stands out for its unique and quite popular flavor. Yet flavor is not only a key component of the sensorial quality of fruits that influences consumer preference; it may also function through olfactory stimuli as a cue to the nutritive value of foods, given that many flavor-related volatiles are derived from essential nutrients (e.g., sugars and lipids) (11, 12). Flavor is a complex combination of volatile and non-volatile components that define aroma, taste, and texture. Non-volatile compounds contribute to the taste of the product. The taste of carob mainly depends on sugars and secondarily on tannins and phenolics (7, 13), while carob aroma is configured by a combination of diverse volatile organic compounds (VOCs) including acids, esters, ketones, aldehydes, alcohols, and furans (14). Volatile compounds are usually present in low concentration, however wide variation may be encountered in the concentrations of individual volatile compounds (15). Each volatile compound has an odor threshold and a sensory descriptor that can be perceived by the human olfactory system (16).

Many factors affect the composition of volatile and non-volatile compounds and consequently the quality of fruits, including maturity stage, genetic background, pre-harvest environment, post-harvest handling, and storage (12, 13, 17, 18). The ripening process is a continuum that comprises the physiological, biochemical, compositional and sensory alternations occurring in the period from fruit setting (anthesis) to senescence (16). The changes in flavor metabolites during fruit development have been extensively studied in different plant species (12, 15). The identity and relative quantity of the accumulating volatiles define the aroma profile of the fruit at progressive stages of ripening. Aromatic compounds usually peak near full fruit maturity; however, the pattern of accumulation and their concentrations differ substantially between plant species. For instance, in prunus fruits, such as apricot and peach, esters tend to peak near full maturity, while 2-hexanal aldehyde that imparts a green tone aroma tends to decline with ripening (19, 20). The composition of taste-linked metabolites such as sugars, phenolics and tannins also changes significantly with the advancement of ripening. In the majority of fruits, soluble sugars reach their highest concentration at the fully ripe stage, whereas organic acids and tannins conversely decline with the progress of ripening, which significantly changes the sweetness, acidity and astringency of the fruit (16).

The effects of ripening on the non-volatile metabolites of the carob pod (pulp) have been recently documented, showing that total phenolic compounds, condensed tannins, organic acids and *in vitro* antioxidant activity decline with ripening, whereas sugar accumulation accelerates with the onset of fruit coloration and peaks at the late ripe stage (13). These changes reduce the astringency and bitterness of the mature carob pulp and enhance favorably its sensory profile. Further to the metabolites that influence taste, volatiles also play a critical role in shaping the overall flavor of the carob pulp. A limited number of studies have been performed to decipher the VOCs profile of the carob intact pod, kibbled pulp or powder, focusing mainly on the aroma characterization of mature carobs from different origins or reporting the effect of roasting on the carob VOCs profile (4, 14, 21–23). The major categories of VOCs detected in mature carob according to the above studies were acids, esters, aldehydes, ketones, alcohols, furans/pyrans, and hydrocarbons. However, the extent to which other factors such ripening, genotype and agro-environmental conditions affect the aroma profile of carob powder and its overall sensorial quality remains largely unstudied. With the increasing demand for sustainable production of foods combining enhanced functional and sensorial properties, carob can be a source of valuable flavor-enhancing components for the food industry. It is therefore imperative to unravel how the aromatic profile of carob powder is configured with respect to the carob fruit's stage of ripening and the effects of other key factors, such as genotype (variety) and agro-environment, potentially contributing to variation in carob flavor.

Accordingly, the overall objective of this study was to examine the configuration of the aromatic profile of carob powder produced from pods harvested at six critical stages of maturity, previously defined based on discriminable physicochemical and physiological characteristics by Kyriacou et al. (13). Moreover, the aroma profile was examined in two distinct genotypic variants of the Cypriot carob (*Ceratonia siliqua* L.) landrace and two altitudinally distinct (15 and 510 m) agro-enviromental zones in order to address how these factors interact with ripening in the configuration of carob powder aroma. The identification of detectible VOCs and the quantification of their relative abundance in carob powder were determined through HS-SPME GC-MS analysis. The current study contributes to our understanding of how the ripening process, genotype and agro-environmental zone interact to configure the volatile aromatic profile of carob powder, which is a potential component of high functional and sensorial value food products.

## Materials and Methods

### Plant Material

**Figure 1:**
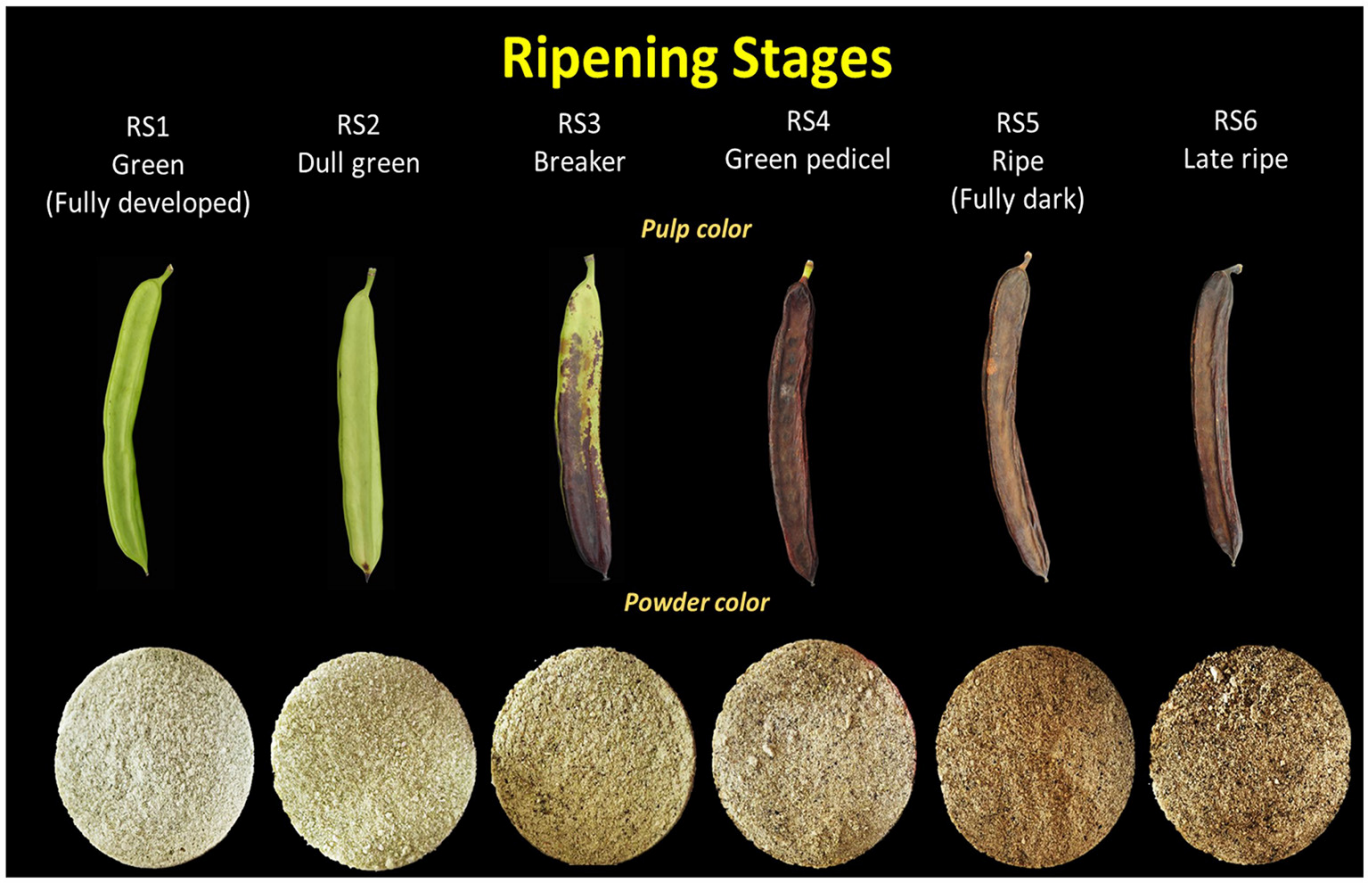
Illustration of intact carob pods and powder color corresponding to six critical fruit ripening stages.

Carob pods were harvested from two genetically similar but phenotypically distinct variants of the Cypriot carob (*Ceratonia siliqua* L.) landrace, known as Lefkaritiki (LF) and Mavroteratsia (MV) (24). Pods were harvested from georeferenced trees that were previously identified genetically. Sampled trees were located at 15 m altitude (coastal zone) in the district of Kalavasos, and at 510 m altitude (mountainous inland zone) in the district of Vavla. Sampling locations were based on previous studies that identified Vavla and Kalavasos as representative of the two major carob agro-environmental zones with respect to pod physicochemical attributes. Three replicate samples of six fruits were harvested at six critical harvest maturity stages (Figure 1; RS1-RS6) (13). Harvest was performed between 08:00 and 10:00 morning hours and the intact carob fruits were subsequently frozen at −40°C. Frozen pods were lyophilized to at 0°C for 48 h in a Christ, Alpha 1-4 lyophilizer (Osterode, Germany) and then coarsely ground in a Vita Prep 3 blender (Vita-Mix Corp., Cleveland, USA) operated at low speed. Deseeded kibbles were further lyophilized to constant weight before being ground to a granulometry of <1 mm in a CT293 Cyclotech mill (Foss Analytical A/S, Hillerød, Denmark). The obtained powder was stored at −60°C for VOCs analysis.

### HS-SPME Volatiles Isolation

The volatile fraction of carob powder was extracted through headspace SPME using a 2 cm Supelco (Park, Bellefonte, Pa., U.S.A.) fiber coated with 50/30 μm Divinylbenzene/Carboxen/ Polydimethylsiloxane (DVB/CAR/PDMS), in combination with gas chromatography/mass spectrometry. Approximately 4 g of carob powder was weighted from each sample and placed in 20 ml clear glass vials (Agilent Technologies Santa Clara, CA, USA). The vials were immediately capped with 20 mm sealing caps using a crimper and incubated at 25°C for 24 h. To enhance volatile emission before adsorption, samples were incubated for 30 min at 50°C (4), then the SPME fiber was inserted into the vial through the septum of the cap and exposed to the headspace of the sample to adsorb volatiles for 30 min (sampling time). Three replicate samples were analyzed for each genotype, location, and ripening stage. Before each run, the SPME fiber was preconditioned at 300°C for 30 min. A system blank injection was performed between samples to check for residual compounds and minimize carryover contamination.

### GC- MS Analysis of VOCs

Volatile compounds analysis was performed on an Agilent 7890A GC system, connected to a 5977B GC/MSD mass selective detector (Agilent Technologies Santa Clara, CA, USA). Separation of compounds was facilitated with a Supelco SPB-624 capillary column of 60 m × 0.25 mm with 1.40 μm film thickness (fused silica). Adsorbed compounds were thermally desorbed at 280°C for 1 min by exposing the SPME fiber into the GC inlet operated in a 1:10 split mode. The GC conditions were based on the methodology of Krokou et al. (14), with inlet temperature set at 280°C and oven temperature starting at 35°C for 5 min, ramped to 180°C at 4°C/min and kept for 10 min. Helium was the carrier gas at a continuous flow rate of 1.7 ml/min. Mass spectrometer was operated in the electron ionization mode (EI) at 70 eV and a spectra range from 35 to 350 m/z. The temperatures set in the MS source, transfer line and quadrupole were 230, 250, and 150°C, respectively. All biological replicates were extracted and analyzed in parallel under identical conditions. The identification of the volatiles was performed by matching their recorded mass spectra with those stored in the NIST17 library of the GC–MS data system. Further verification was made by comparison of their retention indices with external analytical standards including the volatile free acid mixture (Supelco, CRM46975), butyric acid (Sigma-Aldrich, W222208), isobutyric acid, (Sigma-Aldrich, W222119), valeric acid (Sigma-Aldrich, W310107), and acetic acid (Sigma-Aldrich, 695092). The percentage determination was based on peak area normalization without using correction factors.

### Statistical Analysis and Other Software

Data were subjected to three-way analysis of variance (ANOVA). For each of the examined variables the percentage of total variance (PTV) was determined based on the Sum of Squares (SS) [PTV = SS effect/(SS effect + SS error] in order to describe the proportion of the total variance attributed to the main effects and their interactions. Mean comparisons between ripening stages were performed according to Tukey's b multiple range test at a significance level of *p* < 0.05. The hierarchical cluster analysis was performed using the Ward linkage method. Statistical analyses were executed using the SPSS ver. 25 statistical package (IBM, SPSS; Armonk, NY, USA). Spearman's Rho correlations coefficient on the principal VOC groups mean values were calculated to explore relations between VOCs categories. Heatmap was created using the online software DISPLAYR (https://www.displayr.com/).

## Results and Discussion

### Categorical Variation in VOCs Profile at Critical Stages of Carob Ripening

The flavor of fruits and vegetables is configured by both non-volatile and volatile components (15), as both taste and olfaction contribute to the sensory perception of flavor (12). Thus the diverse mixture of volatiles responsible for aroma, also influences the perceived flavor of fruits and vegetables (25). The unique aroma of a mature fruit is usually not evident in the early stages of fruit development but is progressively configured during the process of ripening (16). Likewise, the aroma of ripening carob fruit undergoes substantial and readily perceptible changes emanating from changes in VOCs composition. Such constitutive changes in aroma are also transferred to carob powder (carob flour), which is a primary product of the carob pod milling operation following the deseeding of the pods. Six critical maturity stages were defined and characterized in a previous study based on physiological, morphological, and phytochemical characteristics of the carob pods (13). In the aforementioned work, we demonstrated that the functional (bioactive) quality of the carob pulp is reduced with progressive physiological ripening of the pods, whereas the sensory quality improves to deliver a palatable organoleptic fruity profile at full ripeness.

In the current work, a total of 56 different volatile constituents were detected in carob powder across maturity stages, which amounted to 95-99% of total volatile composition (Table 1). The diversity of volatile components found in carob powder is in accordance to a previous study that recorded 54 volatiles in mature intact carob pods (23). Several factors may affect the efficiency of volatiles detected using HS-SPME-GC-MS methodology, including the VOCs extraction method and the SMPE fiber properties (14). Moreover, thermal processing (roasting) of mature carob pods, which is often applied prior to milling, significantly alters the quantity rather than the quality of the detected volatiles (4). The VOCs presently identified in carob powder at different maturity stages can be distinguished in seven principal groups: acids, esters, aldehydes, ketones, alcohols, furans, and alkanes (Table 1). The same categories of VOCs were identified in previous studies investigating the aromatic profile of mature carobs from different countries (14, 23) and that of roasted vs. un-roasted carobs (4). Remarkably, the terpenoids, which represent the largest and most diverse category of volatiles responsible for the aroma and flavor of many fruits and vegetables, are absent from the carob aromatic profile (15). For example, terpenes and terpenols qualitatively and quantitatively represent the main group of volatiles in orange juice and grapes (27, 28). Lactones is another well-known category of VOCs commonly present in fruits that was not detected in carobs.

**Table 1:** List of VOCs detected in carob powder from all ripening stages, including their retention time, chemical formula, PubChem CID, and a description of their odor.

**Peak**	**RT**	**Name**	**Molecular formula**	**PubChem CID**	Odor description^*^
**ACIDS**					
1	15.841	Acetic acid	C2H4O2	176	sour
2	20.779	Propanoic acid (Propionic acid)	C3H6O2	1032	pungent and unpleasant smell somewhat resembling body odor
3	24.516	Propanoic acid, 2-methyl- (Isobutyric acid)	C4H8O2	6590	acidic sour cheesy dairy buttery rancid
4	25.548	Butanoic acid (Butyric acid)	C4H8O2	264	sharp acetic cheesy buttery fruity (product of anaerobic fermentation)
5	27.960	Butanoic acid, 3-methyl- (Isovaleric acid)	C5H10O2	10430	fruity sweet apple pineapple tutti frutti
6	28.320	Butanoic acid, 2-methyl- (2-methylbutyric acid)	C5H10O2	8314	pungent acidic cheesy roquefort cheese cheesy
7	29.867	Pentanoic acid (Valeric acid)	C5H10O2	7991	acidic sharp cheesy sour milky tobacco fruity
8	34.320	Hexanoic acid (Caproic acid)	C6H12O2	8892	sour fatty sweaty cheesy
**ESTERS**					
9	9.290	Acetic acid, methyl ester	C3H6O2	6584	ethereal sweet fruity
10	13.438	Ethyl Acetate	C4H8O2	8857	ethereal fruity sweet weedy green/ ester
11	17.220	Propanoic acid, 2-methyl-, methyl ester (Methyl isobutyrate)	C5H10O2	7749	fruity green apple pear tart grape berry ripe berry winey peach
12	18.636	Propanoic acid, ethyl ester	C5H10O2	11039	sweet fruity rummy juicy fruity grape pineapple
13	19.300	Butanoic acid, methyl ester	C5H10O2	12180	fruity apple sweet banana pineapple
14	21.132	Butanoic acid, 2-methyl-, ethyl ester	C7H14O2	24020	sharp sweet green apple fruity
15	21.140	Propanoic acid, 2-methyl-, ethyl ester (Isobutyric acid ethyl ester)	C6H12O2	7342	sweet fruity ethereal rummy
16	22.210	Butanoic acid, 2-methyl-, methyl ester	C6H12O2	13357	ethereal estery fruity tutti frutti apple green apple lily of the valley powdery fatty
17	23.400	Butanoic acid, ethyl ester	C6H12O2	7762	fruity green apricot pear banana
18	29.050	Propanoic acid, 2-methyl-, 2-methylpropyl ester	C8H16O2	7351	waxy, green, sweet, orange and aldehydic with vegetative and herbal nuances
19	29.630	Hexanoic acid, methyl ester	C7H14O2	7824	sweet fruity pineapple waxy green banana
20	31.923	Propanoic acid, 2-methyl-, 1-methylbutyl ester	C9H18O2	551303	none found
21	32.950	Hexanoic acid, ethyl ester	C8H16O2	31265	sweet fruity pineapple green peach tropical
22	33.570	Propanoic acid, 2-methyl-, 3-methylbutyl ester (Isoamyl isobutyrate)	C9H18O2	519786	ethereal fruity fruit tropical fruit pineapple grape skin banana
23	33.760	Propanoic acid, 2-methyl-, 2-methylbutyl ester	C9H18O2	97883	fruity ethereal tropical banana
24	39.249	Propanoic acid, 2-methyl-, hexyl ester (Isobutyric hexyl ester)	C10H20O2	16872	sweet, ethereal, fruity, alcoholic fusel rummy
25	39.433	Hexanoic acid, 2-methylpropyl ester	C10H20O2	7775	fruity apple sweet banana pineapple
26	39.861	Butanoic acid, 1-methyl hexyl ester	C11H22O2	529496	green sweet fruity apple waxy soapy
**ALCOHOLS**					
27	7.238	Ethanol	C2H6O	702	strong alcoholic ethereal medical
28	8.802	Isopropyl Alcohol	C3H8O	3776	alcoholic musty woody
29	15.595	1-Propanol, 2-methyl-	C4H10O	6560	ethereal winey
30	18.383	1-Penten-3-ol	C5H10O	12020	ethereal horseradish green radish chrysanthemum vegetable tropical fruity
31	21.380	1-Butanol, 3-methyl- (Isoamyl alcochol)	C5H12O	31260	sweet green fruity apple pineapple nutty
32	28.043	1-Hexanol	C6H14O	8103	ethereal fusel oily fruity alcoholic sweet green
33	29.326	2-Heptanol	C7H16O	10976	fusel alcoholic whiskey fruity banana
34	38.038	2-Nonanol	C9H20O	12367	waxy green creamy citrus orange cheesy fruity
**KETONES**					
35	28.910	2-Heptanone	C7H14O	8051	fruity spicy sweet herbal coconut woody
36	13.027	2,3-Butanedione	C4H6O2	650	buttery sweet creamy pungent caramellic
37	18.151	1-penten-3-one	C5H8O	15394	pungent peppery mustard garlic onion
38	18.294	2-Pentanone	C5H10O	7895	sweet fruity ethereal winey banana woody
39	20.957	Acetoin	C4H8O2	179	pungent sweet creamy buttery
40	27.935	2-Nonanone	C9H18O	13187	fresh sweet green weedy earthy herbal
41	8.388	Acetone	C3H6O	180	alcoholic musty woody
**ALDEHYDES**					
42	11.042	Propanal, 2-methyl- (Isobutyraldehyde)	C4H8O	6561	fresh aldehydic floral pungent
43	16.036	Butanal, 3-methyl-	C5H10O	11552	ethereal aldehydic chocolate peach fatty
44	16.457	Butanal, 2-methyl-	C5H10O	7284	musty chocolate nutty malty fermented
45	16.774	Acetaldehyde	C2H4O	177	pungent ethereal aldehydic fruity
46	18.68	Pentanal	C5H10O	8063	fermented bready fruity nutty berry
47	24.206	Hexanal	C6H12O	6184	fresh green fatty aldehydic grassy leafy fruity sweaty
48	27.660	2-Hexenal	C6H10O	5281168	green banana aldehydic fatty cheesy
49	34.043	Octanal	C8H16O	454	aldehydic waxy citrus orange peel green fatty
50	38.390	Nonanal	C9H18O	31289	waxy aldehydic rose fresh orris orange peel fatty peely
**FURANS**					
51	17.563	Furan, 2-ethyl	C6H8O	18554	sweet burnt earthy malty
52	32.362	Furan, 2-pentyl-	C9H14O	19602	fruity green earthy beany vegetable metallic
**ALKANES**					
53	6.932	Pentane	C5H12	8003	gasoline-like odor^**^
54	9.725	Cyclopentane	C5H10	9253	mild, sweet^**^
55	16.547	Heptane	C7H16	8900	sweet ethereal
56	22.018	Octane	C8H18	356	gasoline

* *Odor description was adopted from the Good Scents Company 2021 [source: (26)], apart from*

** *in which odor description was adopted from the National Library of medicine database (source: https://webwiser.nlm.nih.gov/substance?substanceId)*.

**Figure 2:**
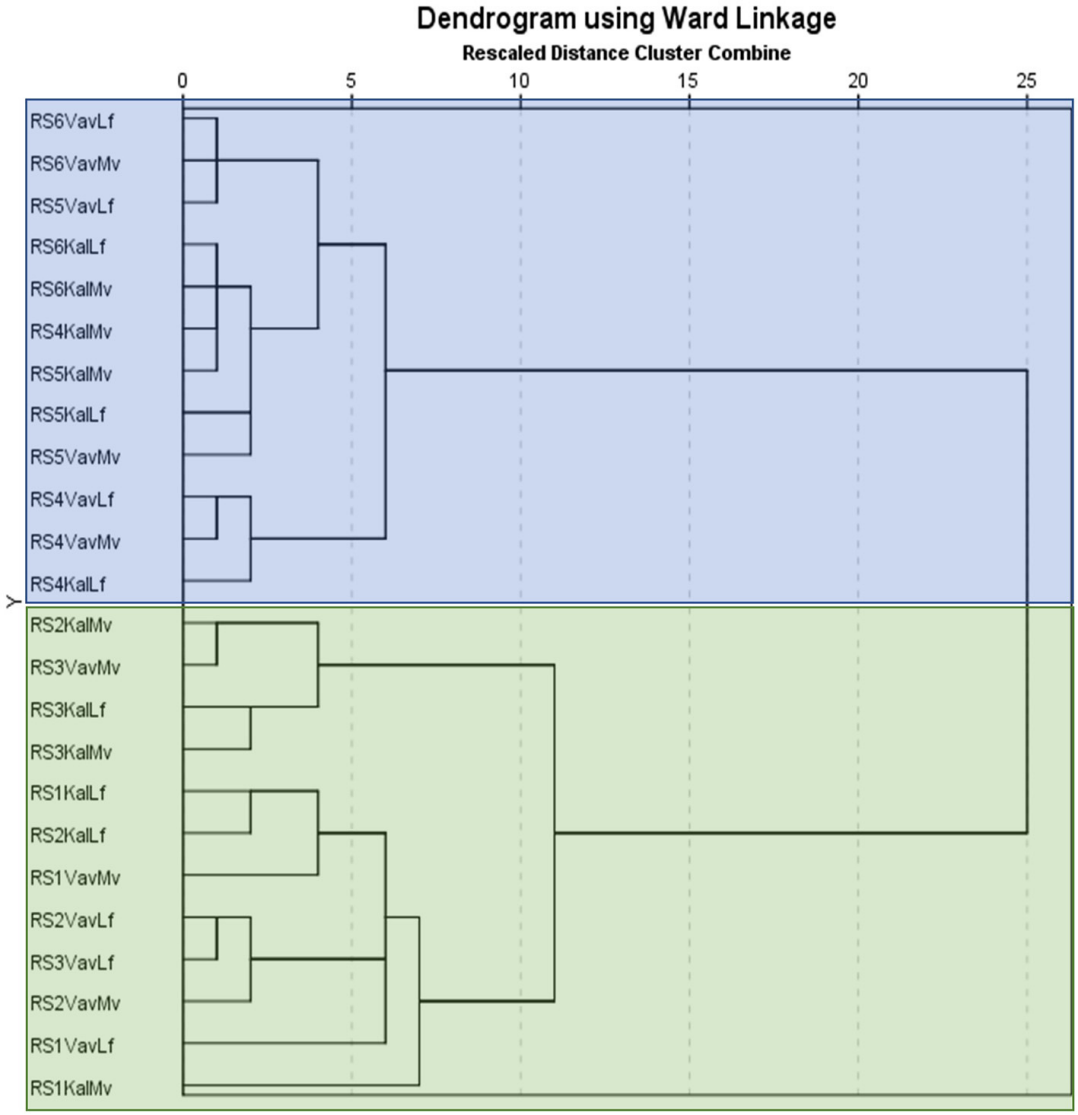
Ward linkage dendrogram of the two genotypes and two agro-enviromental zones (altitudes) at 6 critical ripening stages, based on the detected VOCs. The vertical axis presents the codes of examined samples and the horizontal axis displays the rescaled cluster distance. The first component of the id code refers to ripening stages (RS1-RS6), the second to agro-enviromental zone [Kal, Kalavasos (15 m) and Vav, Vavla (510 m)] and the third to genotype (Mv, Mavroteratsia and Lf, Lefkaritiki). The two major clusters are presented in blue and green transparent boxes.

The GC-MS chromatograms of VOC identified peaks obtained from all the maturity stages examined are demonstrated in Supplementary Figure 1. It is notable that the number of detected compounds increased and their relative abundance changed with progressive ripening until late maturity. It is well-known that although a high number of volatile compounds have been identified in many fruits, only a small fraction of these components have an impact in fruit flavor and aroma based on their quantitative abundance and olfactory threshold (18). The most abundant volatile compound detected throughout carob ripening remained 2-methyl-propanoic acid (isobutyric acid), which contributes definitively to the cheesy-acidic-buttery aroma characteristic of carob. Major changes were revealed through ripening in the constitutive categories of VOCs composing the aroma of carob powder. These changes related both to the composition of the VOC profile and the relative abundance of the detected components. Hierarchical clustering of volatiles composition with ripening stage was performed and the results are presented in dendrogram form (Figure 2). Two main cluster groups were delineated based on the pattern of detected metabolites. The first group consisted of the early stages (RS1 and RS2) and the color breaker stage (RS3) and the second group consisted of the physiologically mature green-pedicel stage (RS4), the fully ripe (RS5) and late-ripe stages (RS6). This cluster pattern indicates that major changes in carob aroma are initiated between the onset of fruit coloration (RS3) and the green pedicel stage (RS4). It is worth noting that major changes in non-aromatic primary and secondary metabolites (e.g., sugars, phenolics, and tannins) were also found to take place during transition to the RS4 stage, which signals the onset of physiological maturity (13).

**Figure 3:**
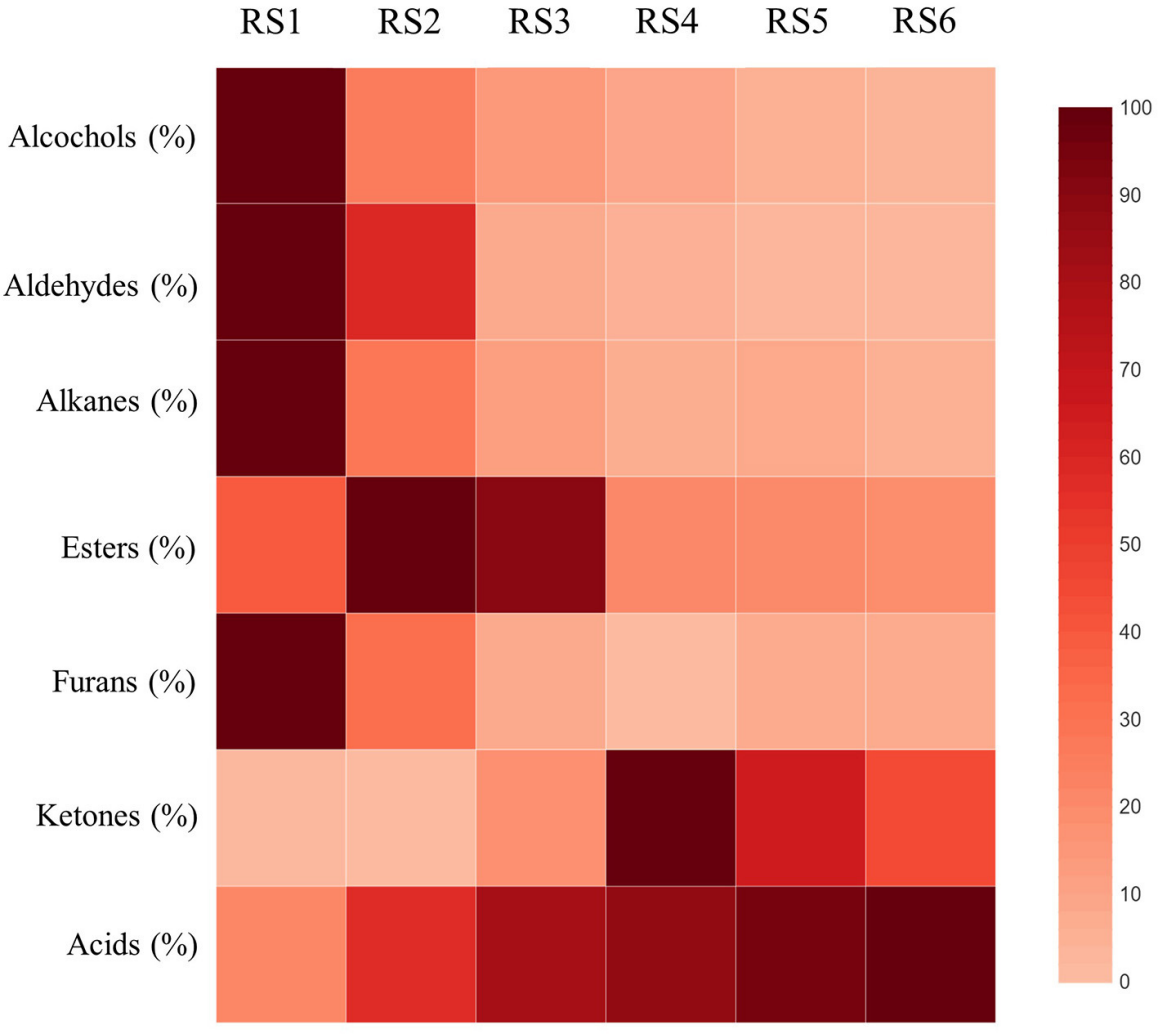
Heatmap illustration of the relative abundance of the seven VOCs categories present in carob powder at the six critical fruit ripening stages (RS1-RS6). Higher abundance presented in darker red color. A scale of color intensity corresponding to relative abundance (0-100 %) is depicted as legend.

Of the three main factors examined (maturity-altitude-genotype) with respect to their effect on the carob powder volatile profile, maturity was the most influential. Maturity is generally one of the most critical factors that influence the fruit metabolome (18). Variation in acids and aldehydes was almost entirely controlled by the ripening stage, which determined 84 and 74% of the total variance for these two VOCs categories, respectively (Table 2). Early ripening stages showed relatively less diverse composition of VOCs, consisting mainly of alcohols, aldehydes, alkanes and furans (Table 2; Figure 3). The powder milled from late mature carob pods carried the full aroma typically associated with carobs, being rich in acids and, to lesser extent, esters. The transition from RS3 (breaker stage) to RS4 (green pedicel stage) triggered substantial changes in the aroma profile (Figure 3). The relative abundance of total acids increased dramatically by almost five-fold between the fully developed unripe (RS1; 19%) and the late ripe stage (88%; RS6, Table 2), with the highest incremental rise in acids occurring in transition from RS1 (19%) to RS2 (51%). Contrarily, the relative abundance of esters, alcohols, aldehydes, alkanes, and furans reduced significantly from RS1 to RS6 (Table 2; Figure 3), with the exception of esters that peaked at RS2 before subsiding and ketones that demonstrated a fluctuated pattern of decline. Concerning the correlations between the major categories of VOCs detected, the highest negative correlations were obtained for aldehydes and alcohols with acids (*r* = −0.835 and *r* = −0.950, respectively; *p* < 0.001). On the other hand, the correlation between aldehydes and alcohols was recorded as the highest positive correlation among the groups of VOCs (*r* = 0.789 and *p* < 0.001; Supplementary Table 1). On the contrary, in apples and strawberries the production of esters increased at late maturity stages (29, 30). Notably, as shown in other studies, the production of C6 compounds including some aldehydes and alcohols increased in early ripening stages and decreased during the progression of ripening (18).

**Table 2:** Analysis of variance, percentage of total variance (PTV) and mean comparisons for total acids, esters, alcohols, ketones, aldehydes, alkanes, and furans of carob pulp powder milled from two genotypes cultivated at two altitudes and harvested at six critical ripening stages.

**Source of variation**	**Acids (%)**		**Esters (%)**		**Alcochols (%)**		**Ketones (%)**		**Aldehydes (%)**		**Furans (%)**		**Alkanes (%)**	
	**PTV**	**Sig**	**PTV**	**Sig**	**PTV**	**Sig**	**PTV**	**Sig**	**PTV**	**Sig**	**PTV**	**Sig**	**PTV**	**Sig**
Maturity (M)	84.1	^***^	43.0	^***^	62.6	^***^	37.6	^***^	78.2	^***^	37.2	^***^	28.9	^***^
Altitude (A)	0.8	^***^	8.3	^***^	0.4	^***^	8.6	^***^	5.8	^***^	1.6	^***^	4.4	^***^
Genotype (G)	0.0	^*^	0.5	^**^	0.3	^***^	3.9	^***^	0.0	ns	1.1	^***^	3.1	^***^
M x A	6.2	^***^	17.4	^***^	15.7	^***^	16.3	^***^	7.8	^***^	2.2	^***^	37.8	^***^
M x G	2.4	^***^	11.2	^***^	2.0	^***^	7.2	^***^	0.1	ns	2.4	^***^	10.5	^***^
A x G	2.8	^***^	5.5	^***^	7.4	^***^	3.0	^**^	1.1	^***^	14.7	^***^	1.2	^***^
M x A x G	3.2	^***^	12.7	^***^	11.1	^***^	22.5	^***^	6.5	^***^	39.5	^***^	13.3	^***^
Error	0.45	1.47	0.53	0.92	0.56	1.36								
Ripening stage	%		%		%		%		%		%		%	
RS1	18.70 g		7.74 c		19.44 a		13.85 a		31.90 a		1.16 a		7.22 a	
RS2	50.52 f		19.73 a		6.88 b		1.60 e		18.88 b		0.37 b		2.02 b	
RS3	72.18 d		17.86 b		3.64 c		3.26 d		2.19 c		0.08 c		0.79 c	
RS4	77.63 c		4.04 d		1.96 d		14.62 a		1.36 c		0.00 c		0.39 c	
RS5	84.60 b		3.89 d		0.95 e		9.35 b		0.59 c		0.07 c		0.56 c	
RS6	88.36 a		3.61 d		0.74 e		6.33 c		0.60 c		0.07 c		0.29 c	
**Altitute**															
Kalavasos (15 m)	67.67 a		12.45 a		5.06 b		5.80 b		5.98 b		0.21 b		2.83 a	
Vavla (510 m)	62.99 b		6.507 b		6.15 a		10.53 a		12.5 a		0.38 a		0.92 b	
***Genotype***															
Lefkaritiki	64.78 b		8.761 b		6.02 a		9.76 a		9.38		0.22 b		1.08 b	
Mavroteratsia	65.88 a		10.20 a		5.18 b		6.58 b		9.12		0.36 a		2.67 a	

*Data represent treatment means of three replicates, each consisting of six fruits. ns, nonsignificant effect;*

* *significant effect at the p < 0.05*,

** *significant effect at the p < 0.01*,

*** *significant effect at the p < 0.001. Means followed by different letters within each column indicate significant differences (between ripening stages, location, genotype) according to Tukey's b-test (p < 0.05)*.

The influence of genotype and environment on the aroma of fruits and vegetables has been previously demonstrated in numerous studies (15, 18, 31). The genetic background showed a significant contribution to the composition and concentration of volatiles in grape and apple cultivars. Terpenoids were in high abundance in *Vitis vinifera* with muscat aroma, while esters were the principal aroma compounds in *V. labrusca* and its hybrids (32). In the apple cultivar “Zaofengtian,” two unique volatiles were detected (β-Damascenone and estragole), which are not abundant in other early apple cultivars (33). The current work showcased agro-environmental zone, expressed principally by altitude, as the second most influential factor affecting volatile composition in carob powder, while the effect deriving from genetic variants of the same landrace (Lefkaritiki and Mavroteratsia) was overall limited. Contrary to our finding, in a recent study of different carob genotypes from Spain, Italy and Cyprus, it was demonstrated that genotype significantly affects the VOCs profile of ground mature carob pods (23).

The predominant carob powder VOCs in terms of relative abundance were acids and esters, which were highest in carobs from low altitudes, while the rest VOCs (alcohols, ketones, aldehydes, and furans) were proportionally more abundant in carobs from higher altitude (Table 2). It is worth noting that the higher mean relative abundance of acids observed in low altitude carobs was related to the accumulation of acids responsible for less pleasant or even unpleasant aroma. The accumulation of free fatty acids resulting from the hydrolysis of triglycerides may lead to further oxidation of fats (hydrolytic rancidity), giving rise to unpleasant off-odors (34). The rancid flavor of oxidized lipids is principally associated with the production of pent-1-en-3-one, oct-1-en-3-one, 2-pentenylfuran and 1-c 5-octadien-3-one (35). Moreover, the dominant aromatic acid responsible for the characteristic carob aroma (isobutyric acid) was relatively more abundant in carobs from the higher altitude (Table 3; Supplementary Figure 2). This finding constitutes a significant indication that carobs cultivated at higher altitudes tend to have more characteristic carob-like aroma and probably better sensory qualities, particularly in terms of aroma. These results corroborate our previous study, which demonstrated that functional and sensory quality were also superior in carobs cultivated inland at mid-altitude compared with those of the coastal zone of cultivation (13). Nevertheless, it should not go unnoticed that the interactions between the main factors examined were also significant; particularly the interaction between location and maturity, which signifies that effect of the ripening process on the configuration of carob powder aroma might present significant variation between different altitudes (Table 2).

**Table 3:** Mean comparisons for the relative abundance of volatile organic acids in the headspace of carob powder milled from two genotypes cultivated at two altitudes and harvested at six critical ripening stages.

	**Acetic acid**	**Propanoic acid**	**Propanoic acid, 2-methyl- (isobutyric acid)**	**Butanoic acid**	**Butanoic acid, 3-methyl-**	**Butanoic acid, 2-methyl- (methylbutyric acid)**	**Hexanoic acid**	**Pentanoic acid**
	**%**	**%**	**%**	**%**	**%**	**%**	**%**	**%**
**Maturity**								
RS1	1.02 b	0.00 d	17.31 d	0.00 e	0.00 c	0.37 g	0.00 e	0.00 c
RS2	0.34 b	0.00 d	48.81 c	0.27 e	0.00 c	1.10 f	0.00 e	0.00 c
RS3	0.54 b	0.07 c	66.09 a	2.71 d	0.00 c	1.70 c	1.07 d	0.00 c
RS4	7.09 a	0.07 c	56.42 b	7.33 c	0.17 b	1.29 d	5.26 c	0.00 c
RS5	7.33 a	0.16 b	56.98 b	9.19 b	0.19 b	1.94 b	8.75 b	0.06 b
RS6	7.67 a	0.18 a	57.73 b	10.18 a	0.21 a	2.15 a	10.16 a	0.08 a
**Location**								
Kalavasos (15 m)	4.35 a	0.11 a	49.75 b	6.33 a	0.10 a	1.95 a	5.04 a	0.04 a
Vavla (150 m)	3.64 b	0.05 b	51.36 a	3.56 b	0.09 b	0.91 b	3.37 b	0.00 b
**Genotype**								
Lefkaritiki	3.96	0.07 b	50.54	4.64 b	0.07 b	1.34 b	4.15	0.02 b
Mavroteratsia	4.04	0.09 a	50.58	5.26 a	0.12 a	1.51 a	4.27	0.03 a

*Data represent treatment means of three replicates, each consisting of six fruits. Means followed by different letters within each column indicate significant differences (between ripening stages, location, genotype) according to Tukey's b-test (p < 0.05)*.

### Acid Components of the Carob Powder VOC Profile

Acid volatiles are the major determinant of the unique cheesy, sour and sweet odor of ripe carobs that is not recorded in other fruits and vegetables. The volatilomes of most well-known and studied fruits such as mango, banana, pineapple, strawberry, pears and citrus are rich in esters, terpenoids and aldehydes, while acids are absent or present in limited abundance (18, 36–39). Acids comprised the most abundant category of the volatile components of the carob powder at all maturity stages except for the immature fully developed green carobs (RS1; Table 2). The relative abundance of acids increased progressively up to late maturity (RS6) to reach 88.4% of the VOCs content. The dominant presence of acids in the VOCs profile of mature carobs has been highlighted also in the recent studies of Krokou et al. (14) and Farag and El-Kersh (4), which reported similar relative abundance of 72-87 and 71-77%, respectively.

Isobutyric acid (propanoic acid 2-methyl) was the dominant volatile component of carob powder from the unripe (17.3%) to the fully mature stage (57.0%). It is a methyl-branched fatty acid also constitutes the main odor-active compound detected in the volatile profile of roasted cocoa powder but in lower (28%) proportion (40). The roasting of carobs was found to reduce the concentration of isobutyric acid in the kibbles to an extent dependent on the roasting temperature and duration (41). Carob powder is considered a possible cocoa substitute as they share the same major volatile compound, however their overall volatile profile is considerably different.

Acetic acid, 2-methyl butanoic acid and 2-methyl propanoic acid (isobutyric acid) were the dominant acids present in immature carobs (RS1-RS3, Table 3). The number of detectible acid volatiles and their relative abundance substantially increased with progressive maturity. Apart from the dominant isobutyric acid, other acids also increased significantly during ripening and contributed considerably to carob aroma. These were butanoic acid and hexanoic acid, both of which reached a relative abundance of 10.2% at RS6, followed by acetic acid with 7.7% and butanoic acid, 2-methyl with 2.2% at RS6. Hexanoic and acetic acids impart sour tones of aroma while butanoic acids configure a more pungent cheesy aroma (Table 1). Similar proportional presence of these respective acids have been reported recently in the aroma profile of intact carob pods obtained from different origins [Krokou et al. (23)]. It is worth noting that some of the acids detected in carob aroma impart unpleasant aromas, such as the propanoic acid that is present in very low percentage but is responsible for what resembles body odor (26). Variation in the acids proportional content were almost entirely controlled by ripening stage (84.1%), with the exception of pentanoic acid and butanoic acid, 2-methyl (Table 2 and Supplementary Table 2). The former was present only in traces at the fully mature stages and was subject to significant interaction of location and maturity stage, while in the case of butanoic acid, 2-methyl location (35.5 %) and maturity stage (45.6%) accounted for most the observed variance (Supplementary Table 1).

### Ester Components of the Carob Powder VOC Profile

Esters constitute a prominent category of VOCs in many fruits and they typically impart a sweet fruity aroma with an ethereal scent in some cases (15, 18). It was also the second most abundant category of volatile compounds present in carob powder, with 18 ester compounds detected across all stages of maturity (Tables 1, 4, 5). The majority of the determined esters were previously detected in the aroma of mature carob pods of different origins (23). Ripening stage and agro-enviromental zone (altitude) explained most of the variance observed in nearly all detected esters (Table 2). During the advancement of ripening, the relative abundance of esters in total showed a significant 2.5-fold increase from RS1 (7.7%) to RS2 (19.7%) and a smaller, however significant, decline at RS3 (17.9%). The turning point for the proportional content of esters was the transition from the breaker stage (RS3) to the green pedicel stage (S4) during which the mean proportional content of esters declined considerably, from 17.9 to 4.0%. Thereafter, from RS4 to RS6, the percentage of esters in the total VOC content remained stable. It is noteworthy that the abundance of esters was two times higher in carobs from low altitudes compared with high altitudes, which parallels the variation observed in the relative abundance of total acids (Table 1).

**Table 4:** Mean comparisons for the relative abundance of volatile esters in the headspace of carob powder milled from two genotypes cultivated at two altitudes and harvested at six critical ripening stages.

	**Acetate**			**Butanoate**						**Hexanoate**			
	**Ethyl acetate**	**Acetic acid, methyl ester**	**Acetate esters**	**Butanoic acid, 2-methyl-, methyl ester**	**Butanoic acid, 2-methyl-, ethyl ester**	**Butanoic acid, ethyl ester**	**Butanoic acid, methyl ester**	**Butanoic acid, 1-methyl, hexyl ester**	**Butanoate esters**	**Hexanoic acid, 2-methylpropyl ester**	**Hexanoic acid, ethyl ester**	**Hexanoic acid, methyl ester**	**Hexanoate esters**
	**%**	**%**	**%**	**%**	**%**	**%**	**%**	**%**	**%**	**%**	**%**	**%**	**%**
**Maturity**													
RS1	0.38 b	0.70 a	1.08 a	0.10 b	0.18 b	0.00 c	0.00 e	0.00 c	0.98 c	0.00 c	0.00 e	0.00 d	0.00 d
RS2	0.40 b	0.00 d	0.4 b	0.23 a	0.76 a	0.20 b	0.04 d	0.00 c	1.23 b	0.00 c	0.00 e	0.00 d	0.00 d
RS3	1.01 a	0.06 c	1.07 a	0.02 c	0.65 a	1.14 a	0.26 a	0.00 c	2.13 a	0.00 c	0.59 a	0.23 c	0.82 a
RS4	0.24 c	0.04 c	0.28 b	0.00 c	0.04 bc	0.22 b	0.12 c	0.06 b	0.48 d	0.00 c	0.39 b	0.31 b	0.70 b
RS5	0.11 d	0.05 c	0.16 c	0.00 c	0.00 c	0.12 b	0.06 d	0.16 a	0.39 d	0.05 a	0.23 c	0.23 c	0.51 c
RS6	0.00 e	0.09 b	0.09 c	0.00 c	0.00 c	0.00 c	0.18 b	0.16 a	0.43 d	0.03 b	0.15 d	0.53 a	0.71 b
**Location**													
Kalavasos (15 m)	0.65 a	0.12 b	0.77 a	0.12 a	0.41 a	0.42 a	0.19 a	0.05 b	1.29 a	0.03 a	0.29 a	0.24 a	0.55 a
Vavla (150 m)	0.07 b	0.20 a	0.27 b	0.00 b	0.13 b	0.14 b	0.03 b	0.08 a	0.58 b	0.00 b	0.16 b	0.20 b	0.35 b
**Genotype**													
Lefkaritiki	0.58 a	0.10 b	0.68 a	0.08 a	0.19 b	0.24 b	0.08 b	0.05 b	0.74 b	0.03 a	0.23	0.17 b	0.42 b
Mavroteratsia	0.13 b	0.22 a	0.35 b	0.04 b	0.35 a	0.32 a	0.14 a	0.08 a	1.14 a	0.00 b	0.23	0.26 a	0.49 a

*Data represent treatment means of three replicates, each consisting of six fruits. Means followed by different letters within each column indicate significant differences (between ripening stages, location, genotype) according to Tukey's b-test (p < 0.05)*.

**Table 5:** Mean comparisons for the relative abundance of propanoate esters in the headspace of carob powder milled from two genotypes cultivated at two altitudes and harvested at six critical ripening stages.

	**Propanoic acid, 2-methyl-, 2-methylpropyl ester**	**Propanoic acid, 2-methyl-, 2-methylbutyl ester**	**Propanoic acid, ethyl ester**	**Propanoic acid, 2-methyl-, 3-methylbutyl ester (isoamyl isobutyrate)**	**Propanoic acid, 2-methyl-, ethyl ester (Isobutyric ethyl ester)**	**Propanoic acid, 2-methyl-, hexyl ester (isobutyric hexyl ester)**	**Propanoic acid, 2-methyl-, 1-methylbutyl ester**	**Propanoic acid, 2-methyl-, methyl ester (Methyl isobutyrate)**	**Propanoate esters**
	**%**	**%**	**%**	**%**	**%**	**%**	**%**	**%**	**%**
**Maturity**									
RS1	0.00 e	0.00 d	0.00 c	0.00 d	3.88 b	0.00 d	0.00 b	2.50 b	6.38 c
RS2	0.00 e	0.00 d	0.05 b	0.00 d	10.99 a	0.00 d	0.00 b	7.06 a	18.10 a
RS3	0.81 a	0.00 d	0.13 a	0.07 d	11.69 a	0.00 d	0.00 b	1.21 c	13.91 b
RS4	0.37 c	0.12 c	0.00 c	0.77 c	1.03 c	0.05 c	0.00 b	0.29 d	2.63 d
RS5	0.60 b	0.31 b	0.00 c	1.24 b	0.39 c	0.11 b	0.09 a	0.15 d	2.88 d
RS6	0.28 d	0.37 a	0.00 c	1.53 a	0.16 c	0.16 a	0.00 b	0.17 d	2.65 d
**Location**									
Kalavasos (15 m)	0.50 a	0.16 a	0.04 a	0.61	6.32 a	0.05 b	0.02 a	2.26 a	9.96 a
Vavla (150 m)	0.18 b	0.11 b	0.02 b	0.60	3.06 b	0.06 a	0.01 b	1.53 b	5.56 b
**Genotype**									
Lefkaritiki	0.50 a	0.12 b	0.02 b	0.53 b	3.76 b	0.06	0.01 b	2.05 a	7.02 b
Mavroteratsia	0.19 b	0.15 a	0.05 a	0.68 a	5.63 a	0.05	0.02 a	1.75 b	8.49 a

*Data represent treatment means of three replicates, each consisting of six fruits. Means followed by different letters within each column indicate significant differences (between ripening stages, location, genotype) according to Tukey's b-test (p < 0.05)*.

The grouping of esters is based on their carboxylic acids: (i) acetate, (ii) propanoate, (iii) butanoate, and (iv) hexanoate. The current work demonstrated that propanoate esters are the most abundant group present in the carob powder VOC profile, with propanoic acid being the dominant acid in eight ester compounds detected, followed by butanoate, hexanoate, and acetate esters (Tables 4, 5). This is in contrast to the VOC profile of most fruits, in which butanoate and acetate are the main ester groups that configure the fruit aroma (18). Propanoic acid, 2-methyl-, hexyl ester (isobutyric hexyl ester) and propanoic acid, 2-methyl-, methyl ester (methyl isobutyrate) were the two most abundant odor-active esters, with peak levels attained at RS2 and RS3, respectively, and thereafter declining until RS6 (Table 5). Isobutyric hexyl ester imparts an ethereal, alcoholic and rummy odor, while methyl isobutyrate a fruitier odor with tones of green apple, pear, grape, berry and peach (26). Isobutyrate has been detected as one of the most odor-active esters contributing to the aroma of pineapple, which is overall dominated by esters that account for 90% of the total VOC composition (42). It is noteworthy that a pleasant fruity odor, rich in apple tones that remind baby cereal fruit cream, was sensorially observed in carob powders milled at RS2 and especially RS3. This pleasurable characteristic aroma is probably a result of the accumulation of the isobutyrate and methyl isobutyrate esters. According to a previous study, RS2 and RS3 carob powder has a high functional quality profile due to accumulation of phenolics, tannins and other antioxidants that were responsible for an intensely astringent taste (13). The current findings corroborate that the potential value of unripe green carob powder as an admixture component for improving the aroma and functional properties of up-coming health-promoting food products warrants further investigation.

Acetate esters generally impart an ethereal-sweet-fruity aroma (Table 1). In the carob powder headspace, they were detected in moderate abundance (19.8-17.9%) at the dull green and breaker stages of pod maturity (RS2 and RS3), after which they subsided to a steady low (4.0-3.6%) through late maturity (Table 4). Hexanoate esters, which provide tones of tropical aroma rich in banana and pineapple odors (Table 1), were first detected at the breaker stage but remained low in relative abundance up to late maturity. Butanoic acid esters were detected throughout all stages of maturity and peaked at the breaker stage, although their relative abundance in total remained low (<3.0 %) throughout ripening. Ethyl ester and butanoic acid, 2-methyl-, ethyl ester were the two most abundant butanoic acid esters, accountable for green apple and green apricot tones of aroma, respectively (Table 4). Butanoate and acetate esters have been identified in banana, melon, apple and pear varieties where they play a substantial role in the configuration of fruit aroma (39, 43–47). Ethyl 2-methyl butanoate and 2-methyl butylacetate are the main butanoate esters responsible for “Fuji” apples aroma, while ethyl butanoate and ethyl 2-methyl butanoate are the odor-active compounds in “Elstar” apples (48). Acetate is the main group of esters in melon, with 37% of the total volatiles represented by ethyl 2-methyl propyl acetate and 2-methyl butyl acetate (45). The concentration of acetates and butanoates was shown increase during the ripening of banana fruit (44), in contrast to the current findings on carob powder aroma, which registered a general decline of those esters with the advancement of ripening. As for hexanoates, hexanoic acid, ethyl ester was detected in pear and pineapple (42, 43), and according to our findings it is also present in the carob powder volatile profile from the breaker onwards.

### Aldehydes, Alcohols and Furans Composition at Critical Ripening Stages

The variation observed in aldehydes, alcohols and furans was mainly defined by the ripening stage, with a very low contribution deriving from the effects of location and genotype (Table 2). Furans demonstrated lower relative abundance in the carob volatile profile at all stages of maturity compared to the other VOCs categories (Table 2). Aldehydes constitute one of the most abundant categories of VOCs, which encompasses numerous compounds contributing to the aroma of fruits such as stone fruits, apple, strawberries oranges and raspberries but also to the flavor of vegetables such as tomato, cucumber and spinach (15, 49). Aldehydes and alcohols accumulated in green fully developed carobs (RS1) and decreased during the advancement of ripening, which led to the attenuation of the associated green grassy aroma (Figure 3). Significant correlation of alcohols with aldehydes abundance (*r* = 0.789; *p* < 000.1) was reported. Hexanal (16%), butanal, 3-methyl (8%), butanal, 2-methyl (3%), and propanal, 2-methyl (3%) were the main aldehydes with substantial contribution to the total volatilome of unripe carobs at RS1 (Table 6). The abundance of all of the above-mentioned volatiles remained stable up to RS2, with the exception of hexanal, which decreased significantly by around five-fold during the transition from RS1 (16%) to RS2 (3%). Six-carbon odor-active aldehydes with prominent presence at the unripe stages RS1 and RS2, such as hexanal and 2-hexanal, are responsible for fresh green and grassy leafy notes generally associated with green unripe fruits (16). Similar observations were reported with respect to the volatile profile of immature apricot, most abundant in aldehydes, including hexanals, that significantly decreased during fruit ripening (50). Notably, butanal and floral aldehydes (e.g., propanal, 2-methyl) contributed chocolate and floral tones to the overall aromatic profile at RS1 and RS2 that partly explains the pleasurable aroma observed in powder milled from carob pods harvested at these immature stages (Table 1).

**Table 6:** Mean comparisons for the relative abundance of aldehydes in the headspace of carob powder milled from two genotypes cultivated at two altitudes and harvested at six critical ripening stages.

	**Propanal, 2-methyl- (isobutyraldehyde)**	**Butanal, 2-methyl-**	**Butanal, 3-methyl-**	**Acetaldehyde**	**Hexenal**	**2-Hexanal**	**Pentanal**	**Octanal**	**Nonanal**
	**%**	**%**	**%**	**%**	**%**	**%**	**%**	**%**	**%**
**Maturity**									
RS1	2.96 a	3.05 a	8.13 a	0.36 a	15.71 a	1.49 b	0.20 a	0.00 c	0.00 c
RS2	2.87 a	3.06 a	8.13 a	0.0 d	3.05 b	1.77 a	0.00 d	0.00 c	0.00 c
RS3	0.41 b	0.31 b	0.72 b	0.10 bc	0.00 c	0.65 c	0.00 d	0.00 c	0.00 c
RS4	0.26 bc	0.21 b	0.66 b	0.13 b	0.00 c	0.00 d	0.00 d	0.00 c	0.10 b
RS5	0.04 c	0.04 b	0.18 b	0.11 bc	0.00 c	0.00 d	0.08 b	0.02 b	0.11 b
RS6	0.02 c	0.03 b	0.16 b	0.07 c	0.00 c	0.00 d	0.05 c	0.07 a	0.22 a
**Location**									
Kalavasos (15 m)	0.76 b	0.91 b	2.23 b	0.18 a	1.45 b	0.35 b	0.07 a	0.00 b	0.03 b
Vavla (150 m)	1.43 a	1.32 a	3.76 a	0.07 b	4.80 a	0.96	0.04 b	0.03 a	0.12 a
**Genotype**									
Lefkaritiki	1.05	0.99 b	2.65 b	0.07 b	3.85 a	0.68	0.01 b	0.01 b	0.08
Mavroteratsia	1.14	1.24 a	3.35 a	0.19 a	2.41 b	0.63	0.09 a	0.02 a	0.07

*Data represent treatment means of three replicates, each consisting of six fruits. Means followed by different letters within each column indicate significant differences (between ripening stages, location, genotype) according to Tukey's b-test (p < 0.05)*.

Furans are a well-known class of aromatic compounds commanding a substantial role in the configuration of aroma in strawberry, small berries, pineapple and mango (51). According to the current study furans have a minimal contribution in the aroma profile of carob powder. In total, two furans (furan, 2-ethyl and furan, 2-pentyl) were detected, mainly in the carob powder milled from the immature RS1 and RS2 stages, while in the powder milled from mature carobs furans were found in total at trace levels (Table 7), which corroborates previous studies on carob (4, 14, 23). On the contrary, the thermal processing of carob, usually in the form of roasting prior to milling, increased the proportion of furans (from 1.2 to 6.3%) in the total volatile composition and induced the production of pyrans (3.7%), detected almost exclusively in roasted carob (4).

**Table 7:** Mean comparisons for the relative abundance of alcohols and furans in the headspace of carob powder milled from two genotypes cultivated at two altitudes and harvested at six critical ripening stages.

	**Alcochols**								**Furans**	
	**1-Butanol, 3-methyl- (isoamyl alcochol)**	**Ethanol**	**Isopropyl Alcohol**	**1-Propanol, 2-methyl-**	**1-Penten-3-ol**	**1-Hexanol**	**2-Heptanol**	**2-Nonanol**	**Furan, 2-ethyl-**	**Furan, 2-pentyl-**
	**%**	**%**	**%**	**%**	**%**	**%**	**%**	**%**	**%**	**%**
**Maturity**										
RS1	0.00 e	16.12 a	1.55 a	0.00 c	1.77 a	0.00 b	0.00 c	0.00 b	0.57 a	0.59 a
RS2	0.09 b	6.06 b	0.10 b	0.00 c	0.64 b	0.00 b	0.00 c	0.00 b	0.22 b	0.15 b
RS3	0.00 e	3.35 c	0.04 b	0.09 b	0.16 c	0.00 b	0.00 c	0.00 b	0.04 c	0.04 c
RS4	0.30 a	0.89 d	0.04 b	0.19 a	0.00 d	0.10 a	0.44 ab	0.00 b	0.00 c	0.00 c
RS5	0.07 c	0.26 e	0.00 b	0.02 c	0.00 d	0.00 b	0.49 a	0.11 a	0.00 c	0.07 bc
RS6	0.02 d	0.17 e	0.00 b	0.00 c	0.00 d	0.00 b	0.40 b	0.12 a	0.00 c	0.07 bc
**Location**										
Kalavasos (15 m)	0.01 b	4.43	0.10 b	0.04 b	0.30 b	0.02	0.17 b	0.00 b	0.10 b	0.11 b
Vavla (150 m)	0.15 a	4.52	0.48 a	0.06 a	0.56 a	0.02	0.27 a	0.08 a	0.17 a	0.20 a
**Genotype**										
Lefkaritiki	0.07 b	4.75 a	0.35 a	0.05	0.51 a	0.02	0.25 a	0.04 a	0.09 b	0.13 b
Mavroteratsia	0.10 a	4.20 b	0.23 b	0.05	0.35 b	0.02	0.19 b	0.03 b	0.18 a	0.18 a

*Data represent treatment means of three replicates, each consisting of six fruits. Means followed by different letters within each column indicate significant differences (between ripening stages, location, genotype) according to Tukey's b-test (p < 0.05)*.

Alcohols play a minor role in the composition of aroma and flavor of most fruits and vegetables (15). According to our findings, alcohols demonstrated an important contribution to the aroma profile of carob powder at the unripe stages (RS1-19.4% and RS2-6.9%; Table 2). Ethanol was the main contributing alcohol with a significant proportional contribution among alcohols (RS1-16.1% and RS2-6.1%; Table 7) to the aroma profile of unripe carobs, followed by 1-penten-3-ol and isopropyl alcohol. The relative abundance of these key alcohols declined significantly with the advancement of ripening (Table 7). Ethanol is generally not detected in the aromatic profile of other fruits at as high proportion as in unripe carob powder, with exception of avocado. According to El-Mageed (52), ethanol is one of the most abundant volatiles in immature and ripe avocado with a declining tendency as ripening progresses. 2-Heptanol is the only alcohol that appeared after physiological maturity (RS4) and sustained its relative abundance till the over-ripe stage (RS6), although its contribution to the overall volatile composition of carob powder was very low (around 0.4%; Table 7).

### Ketones and Alkanes Composition at Critical Ripening Stages

Ketones were detected in the VOCs profile of carob powder at all ripening stages, and it was the only category that showed great fluctuation throughout ripening (Figure 3). This may be explained by the fact that the control of variation in ketones by maturity was low (37.6% of total variance), while the interactions between the main factors (maturity, altitude, genotype) explained a significant percentage of the variation (49.0%; Table 2). The highest relative abundance of ketones was recorded at RS1 (13.85%) and RS4 (14.62%; Table 2). Acetone was the only ketone detected in high abundance at RS1 and then declined with the progress of ripening (Table 8). Almost all the rest ketones including 2-heptanone, 2,3-butanedione, 2-pentanone, acetoin and 2-nonanone were first detected at RS3 and their abundance increased with the advancement of ripening until RS5, followed by a moderate decline at RS6 (Table 8). These ketones contribute to the carob powder aroma by providing mainly sweet, creamy, woody and pungent tones (Table 1). Apart from carobs, ketones contribute to the aroma of other fruits, such as citrus: grapefruit (1-hepten-3-one) and clemenules (3-pentanone and β-ionone) (28, 53).

**Table 8:** Mean comparisons for the relative abundance of ketones in the headspace of carob powder milled from two genotypes cultivated at two altitudes and harvested at six critical ripening stages.

	**Aceton**	**2-Heptanone**	**2,3-Butanedione**	**1-penten-3-one**	**2-pentanone**	**Acetoin**	**2-Nonanone**
	**%**	**%**	**%**	**%**	**%**	**%**	**%**
**Maturity**							
RS1	13.71 a	0.00 e	0.00 e	0.14 a	0.00 e	0.00 e	0.00 c
RS2	1.60 b	0.00 e	0.00 e	0.00 c	0.00 e	0.00 e	0.00 c
RS3	0.90 bc	1.26 d	0.32 d	0.06 b	0.34 d	0.35 d	0.03 c
RS4	0.89 bc	5.42 a	1.08 a	0.00 c	2.42 a	3.43 a	1.39 a
RS5	0.40 c	4.48 b	0.58 b	0.00 c	1.24 b	1.20 b	1.45 a
RS6	0.28 c	3.01 c	0.39 c	0.00 c	0.61 c	0.86 c	1.23 b
**Location**							
Kalavasos (15 m)	1.12 b	2.48 a	0.31 b	0.05 a	0.800	0.54 b	0.51 b
Vavla (150 m)	4.80 a	2.24 b	0.48 a	0.01 b	0.740	1.41 a	0.86 a
**Genotype**							
Lefkaritiki	4.10 a	2.58 a	0.42 a	0.02 b	0.88 a	1.02 a	0.74 a
Mavroteratsia	1.83 b	2.14 b	0.37 b	0.05 a	0.66 b	0.93 b	0.63 b

*Data represent treatment means of three replicates, each consisting of six fruits. Means followed by different letters within each column indicate significant differences (between ripening stages, location, genotype) according to Tukey's b-test (p < 0.05)*.

Alkanes constitute a family of nearly odorless volatile compounds known to be part of the cuticle wax of leaves and fruits (54–56). The cuticle of sweet cherries is composed by 19% of alkanes that are positively correlated with the tolerance to cracking (56). Waxy cuticle is commonly present on the upper surface of the carob leaflet and its accumulation increases under dry conditions so as to reduce cuticular permeability and water loss (57). According to our findings, alkanes are present in the VOCs profile of unripe carob powder, and this is probably related to the heavily waxy surface of the pod observed at the early stages of ripening. The relative abundance of alkanes declined with progressive ripening but in an altitude-specific manner owing to significant maturity-altitude interaction (Figure 3; Table 2). Genotype and location had a minimal effect on alkanes variation (Table 2). The most representative alkanes were cyclopentane and heptane with their highest abundance recorded in carob powder derived from green immature pods at RS1 (Supplementary Table 3).

## Conclusion

Of the three main factors examined with respect to the carob powder volatile profile, maturity was the most influential, followed by altitude, while the effect of genotype was limited. Major changes in aroma were initiated from the onset of fruit coloration to the green pedicel stage. Fifty-six volatile compounds were identified across maturity stages, encompassing acids, esters, aldehyde, ketones, alcohols, furans and alkanes. The profile of early ripening stages consisted mainly of alcohols, aldehydes, alkanes and furans, whereas acids increased with ripening and dominated the carob powder volatilome at full maturity. Concerning the correlations between the major VOCs categories, aldehydes and alcohols yielded the highest negative correlations with acids, whereas aldehydes and alcohols were the groups presenting the highest positive correlation. The most abundant VOC throughout ripening was isobutyric acid, responsible for the characteristic cheesy-acidic-buttery aroma of carob. Propanoate esters were the most abundant esters, whereas butanoate and acetate esters dominate in most fruits. Aldehydes and alcohols decreased with ripening, resulting in the attenuation of green grassy aroma. A pleasurable aroma was detected at the immature stages, attributed to isobutyrate and methyl isobutyrate esters. The current findings corroborate that unripe green carob powder warrants further investigation as a potential admixture component for improving the aroma profile of novel food products. Total acids and esters were highest at low altitude, but this related mainly to acids of less pleasant aroma. Most importantly, the dominant isobutyric acid was more abundant at higher altitude, which constitutes a significant indication that cultivation at higher altitudes enhances the characteristic carob-like aroma and sensory qualities of carob powder. Future work, should entail the quantification of presently identified key odor-active compounds coupled to sensory analysis in order to establish their olfactory threshold and overall impact on the aroma profile of carob powder.

## Data Availability Statement

The raw data supporting the conclusions of this article will be made available by the authors, without undue reservation.

## Author Contributions

MK, AK, and CA: conceptualization, methodology, and writing—original draft preparation. AK, MK, CA, GS, and YR: formal analysis. MK and YR: resources. CA, AK, and GS: data curation. MK, CA, AK, and YR: writing—review and editing. MK and AK: project administration. All authors have read and agreed to the published version of the manuscript.

## Funding

This research was co-funded by the European Regional Development Fund and the Republic of Cyprus through the Cyprus Research and Innovation Foundation (Project: BlackGold INTEGRATED /0916/0019).

## Conflict of Interest

The authors declare that the research was conducted in the absence of any commercial or financial relationships that could be construed as a potential conflict of interest.

## Publisher's Note

All claims expressed in this article are solely those of the authors and do not necessarily represent those of their affiliated organizations, or those of the publisher, the editors and the reviewers. Any product that may be evaluated in this article, or claim that may be made by its manufacturer, is not guaranteed or endorsed by the publisher.
