# Supplementary material for: Configuration of the Volatile Aromatic Profile of Carob Powder Milled From Pods of Genetic Variants Harvested at Progressive Stages of Ripening From High and Low Altitudes
Source: Front Nutr. 2021 Dec 15;8:789169. doi: 10.3389/fnut.2021.789169 (PMC8714772; doi:10.3389/fnut.2021.789169)
Supplement: Supplementary file 1 [file Data_Sheet_1.docx]

**Supplemental material**

**Supplemental Figure 1.** Indicative HS-SPME-GC-MS chromatogram of each ripening stage: RS1 (A), RS2 (B), RS3 (C), RS4 (D), RS5 (E), RS6 (F). Retention times of the detected VOCs are available in Table 1. The highest peak in all chromatograms corresponds to isobutyric acid, which was the dominant volatile detected in carob powder throughout ripening.


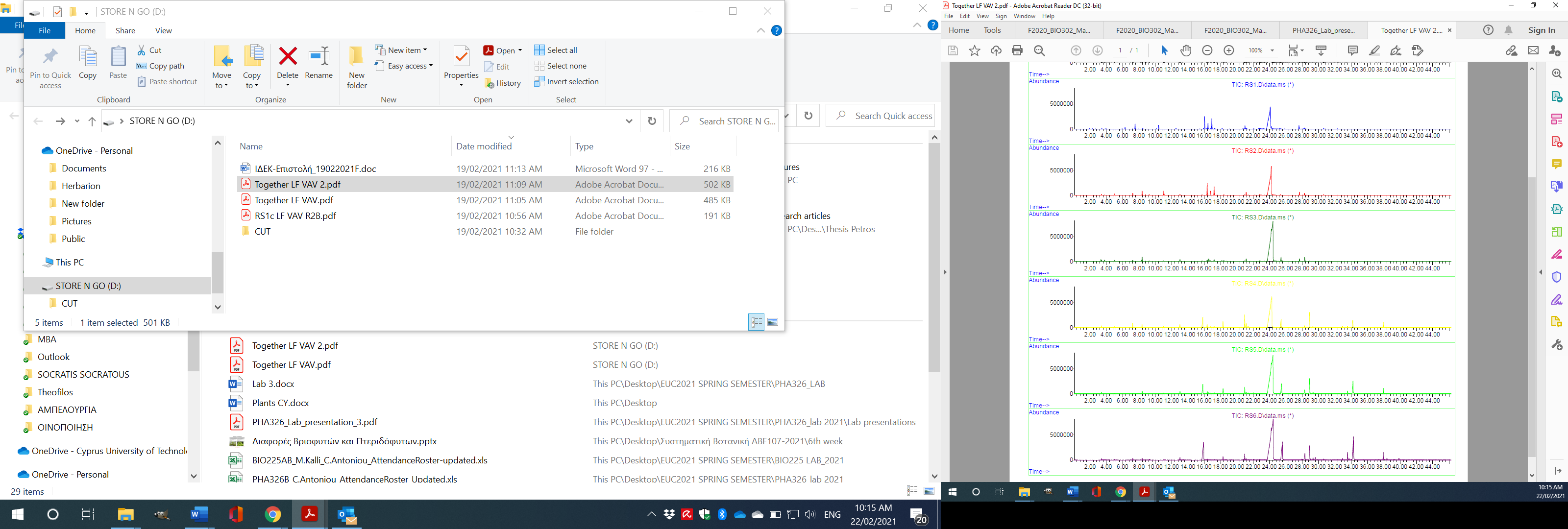


A.

B.

C.

F.

E.

D.

**Supplemental Figure 2.** Interaction plots on mean values of two genotypes (Lafkaritiki and Mavroteratsia) and two locations (Kalavasos-15 m and Vavla-510 m) at ripening stage 6 (RS6) are presented for selected acid volatiles: propanoic acid, 2-methyl (A), acetic acid (B), butanoic acid (C), butanoic acid, 2-methyl (D), butanoic acid, 3-methyl (E), hexanoic acid (F), propanoic acid (G).


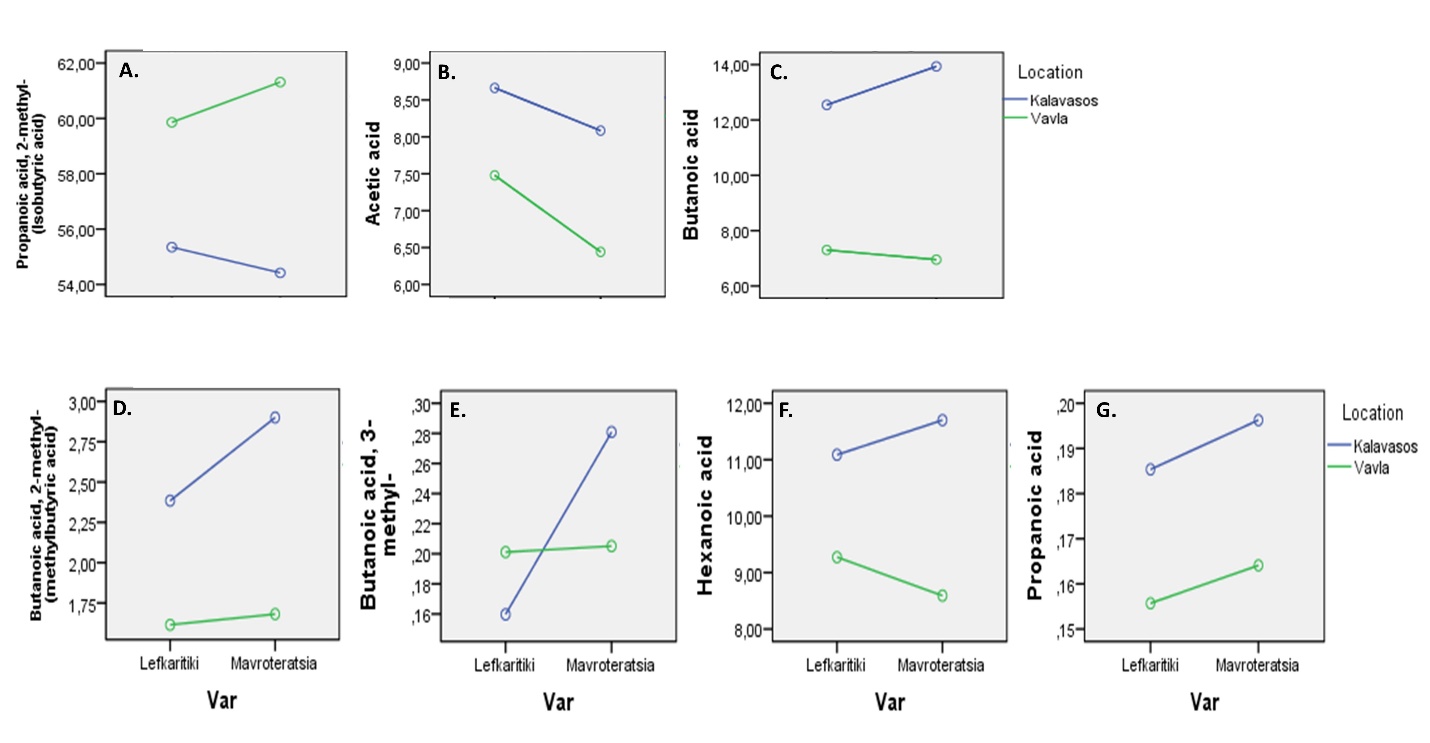


**Supplemental Table 1.** Spearman's Rho correlation coefficients on the major VOC groups mean values.

** Correlation is significant at the 0.01 level (2-tailed)

* Correlation is significant at the 0.05 level (2-tailed)

**Supplemental Table 2.** Analysis of variance and percentage of total variance (PTV) for volatile organic acids of carob pulp powder milled from two genotypes (Lefkaritiki and Mavroteratsia) cultivated at two altitudes (15 and 510 m) and harvested at six critical ripening stages (RS1-RS6).

Data represent treatment means of three replicates, each consisting of six fruits. ns = nonsignificant effect; * significant effect at the p < 0.05, ** significant effect at the p < 0.01, *** significant effect at the p < 0.001.

**Supplemental Table 3.** Mean comparisons for the relative abundance of alkanes in the headspace of carob powder milled from two genotypes (Lefkaritiki and Mavroteratsia) cultivated at two altitudes (15 and 510 m) and harvested at six critical ripening stages (RS1-RS6).

Data represent means of three replicates, each consisting of six fruits, for each treatment. Means followed by different letters within each column indicate significant differences according to Tukey’s b test (p < 0.05).
